# Infectious Bronchitis Virus Infection Induces Ferroptosis-Associated Changes with Lipid Peroxidation Dysregulation and Modulates Viral Replication

**DOI:** 10.3390/biology15201856

**Published:** 2026-10-15

**Authors:** Xiaoxiao Han, Jiaxin Wang, Die Yang, Qifan Zhao, Shuhan Zhang, Jing Zhou, Yanzi Yu

**Affiliations:** 1 School of Bioscience and Technology, Chengdu Medical College, Chengdu 610500, China; 2 Sichuan Higher Education Institute Key Laboratory of Major Disease Target Discovery and Protein Drug Development, Chengdu 610500, China; 3 Experimental Teaching Center of Biotechnology, Chengdu Medical College, Chengdu 610500, China

**Keywords:** chicken macrophage, redox homeostasis, iron metabolism, nucleocapsid protein, epigallocatechin gallate

## Abstract

Infectious bronchitis virus (IBV) causes significant disease in poultry; however, whether ferroptosis-associated responses are involved in IBV-induced cellular injury remains unclear. In this study, we examined ferroptosis-associated changes in the HD11 chicken macrophage cell line following IBV infection. IBV infection reduced cell viability, increased lactate dehydrogenase release, caused mitochondrial ultrastructural alterations, enhanced lipid peroxidation, depleted glutathione, increased intracellular ferrous iron, and altered the expression of ferroptosis-related genes. Treatment with ferrostatin-1 attenuated these changes and was accompanied by reduced viral replication. By contrast, ultraviolet-inactivated IBV did not induce comparable ferroptosis-associated changes, supporting an association between active IBV infection and these responses. Expression of the IBV nucleocapsid protein alone partially reproduced several ferroptosis-associated changes in HD11 cells. Epigallocatechin gallate reduced IBV-induced cellular injury and ferroptosis-associated changes, whereas RSL3 partly reversed its protective effect. Taken together, these results indicate that IBV infection induces ferroptosis-associated changes in chicken macrophages in association with increased lipid peroxidation, while Fer-1 treatment was accompanied by reduced viral replication. These findings support the involvement of ferroptosis-associated responses in IBV-host cell interactions.

## Simple Summary

Infectious bronchitis is an important viral disease of chickens and can affect the respiratory system, kidneys, and egg production. Although vaccines are available, the disease remains a challenge for poultry production. In this study, we used chicken macrophage cells grown in the laboratory to examine how infectious bronchitis virus damages host cells. Macrophages are immune cells that help defend the body against infection. We found that the virus induced ferroptosis-associated changes, including changes related to iron accumulation and oxidative damage to fats in cell membranes. Treatment with a ferroptosis inhibitor reduced cell injury and also lowered virus production. The viral nucleocapsid protein alone also induced changes associated with ferroptosis in chicken macrophages. In addition, epigallocatechin gallate, a natural compound found in green tea, protected the cells from IBV-induced cellular injury and reduced changes associated with ferroptosis. These findings improve our understanding of IBV infection and suggest ferroptosis-associated processes may be involved in virus-induced cellular damage.

## 1. Introduction

Infectious bronchitis virus (IBV), a member of the genus *Gammacoronavirus*, is a highly contagious avian coronavirus that causes respiratory, renal, and reproductive diseases in chickens and results in substantial economic losses to the poultry industry worldwide through mortality, reduced growth performance and egg production, and disease-control costs [1,2]. IBV is mainly transmitted through respiratory secretions, direct contact, and contaminated materials, and mortality varies depending on the viral strain, bird age, immune status, and secondary infections [1,2,3]. IBV primarily infects the respiratory tract but can also spread to the kidneys and reproductive organs, leading to nephritis, reduced egg production, growth retardation, and increased mortality [3]. IBV is characterized by a high mutation and recombination rate, which contributes to the continuous emergence of novel variants. Major IBV lineages include Massachusetts (GI-1), 793B/4/91 (GI-13), QX-like (GI-19), and variant 2 (GI-23), although their prevalence varies geographically [2,4]. As a result, cross-protection among different antigenic types is limited, making disease control more challenging [4,5]. Although live attenuated and inactivated vaccines are widely used to control infectious bronchitis, protection against antigenically different strains is often incomplete [5,6]. Therefore, a better understanding of the interaction between IBV and host cells is important for studying viral pathogenesis and developing effective control strategies.

Programmed cell death is an important host defense mechanism against viral infection and plays a critical role in regulating viral replication, inflammatory responses, and tissue injury [7,8]. Unlike apoptosis, which is characterized by caspase activation, chromatin condensation, and DNA fragmentation, ferroptosis is an iron-dependent form of regulated cell death that occurs independently of the classical apoptotic pathway [9]. Ferroptosis is a newly recognized form of regulated cell death characterized by iron-dependent lipid peroxidation, glutathione depletion, redox imbalance, and typical mitochondrial morphological changes, including mitochondrial shrinkage and loss of cristae [10,11]. Increasing evidence suggests that ferroptosis and ferroptosis-related pathways are involved in various viral infections and may influence virus–host interactions [12,13,14]. In addition, abnormal iron metabolism and excessive lipid peroxidation have been closely associated with virus-induced tissue injury [15]. These findings suggest that ferroptosis may represent an important cellular response during viral infection, but its role may vary depending on the virus and host cell type.

Macrophages are important cells of the innate immune system and participate in antiviral responses, inflammatory regulation, and the clearance of invading pathogens [16]. The chicken macrophage cell line HD11 has been used in several studies as an in vitro model to investigate IBV-macrophage interactions. IBV infection has been shown to alter macrophage viability, phagocytic activity, inflammatory responses, and immune-related gene expression in HD11 cells and chicken primary macrophages [17]. HD11 cells have also been used to study IBV replication, viral regulation of the JAK-STAT pathway, inflammatory responses, and different forms of regulated cell death [18,19,20]. These findings support the use of HD11 cells for mechanistic studies of IBV infection in chicken macrophages. In our previous studies using this model, we found that IBV infection induced ROS-mediated mitochondrial apoptosis and NLRP3 inflammasome-dependent pyroptosis [21,22]. These findings indicate that IBV infection induces oxidative stress and multiple forms of regulated cell death in chicken macrophages. Since oxidative stress, lipid peroxidation, and disturbed redox homeostasis are closely associated with ferroptosis, it is reasonable to investigate whether ferroptosis also contributes to IBV-induced cell injury [9]. However, whether IBV infection induces ferroptosis and whether this process affects viral replication remain unclear. Epigallocatechin gallate (EGCG), a major polyphenol in green tea, has been reported to exert antiviral activity against several viruses, including avian viruses [23]. In addition, EGCG can reduce oxidative damage, lipid peroxidation, and ferroptosis-associated injury [24]. These properties prompted us to further investigate whether EGCG could protect HD11 cells against IBV-induced ferroptotic injury.

In the present study, we hypothesized that IBV infection induces ferroptosis-associated changes in HD11 chicken macrophages and that these changes are associated with viral replication. To test this hypothesis, we examined lipid peroxidation, glutathione homeostasis, intracellular Fe^2+^ levels, ferroptosis-related gene expression, and cellular injury following IBV infection. Fer-1 and UV-inactivated IBV were used to further examine the association between ferroptosis-associated changes, active viral infection, and viral replication. We also investigated whether expression of the IBV N protein alone could induce similar changes and evaluated the effects of EGCG on IBV-induced cellular injury and ferroptosis-associated responses. In this study, MDA, the GSH/GSSG ratio, intracellular Fe^2+^ levels, and viral replication measurements were used as the primary readouts, whereas cell viability and LDH release were used as secondary indicators of cellular injury, with ferroptosis-related gene expression and TEM observations providing supporting evidence. These analyses were designed to characterize the ferroptosis-associated response during IBV infection without excluding the possible involvement of other regulated cell-death pathways.

## 2. Materials and Methods

### 2.1. Cells and Viruses

The chicken macrophage cell line HD11 was purchased from Shanghai Jinyuan Biotechnology Co., Ltd. (Shanghai, China), with cell-line authentication performed by the supplier, and was subsequently maintained in our laboratory. The cells were routinely tested for mycoplasma contamination and were confirmed to be negative during the experiments. The Beaudette strain of infectious bronchitis virus (GenBank: DQ001339) and Vero cells were kindly provided by Prof. Hongning Wang (Sichuan University, Chengdu, China). HD11 cells were cultured in Dulbecco’s modified Eagle’s medium (DMEM; HyClone, Logan, UT, USA) supplemented with 10% fetal bovine serum (Nanjing SenBeiJia Biological Technology, Nanjing, China), 100 U/mL penicillin, and 100 μg/mL streptomycin in a humidified incubator at 37 °C with 5% CO_2_. Viral titers were determined using the median tissue culture infectious dose (TCID_50_) method according to the Reed–Muench method [25]. Briefly, samples were serially diluted 10-fold and inoculated into Vero cells in eight replicate wells per dilution. After incubation for 5–7 days, wells were scored as positive or negative based on the presence or absence of characteristic cytopathic effects (CPE), and viral titers were expressed as log_10_ TCID_50_/mL. For infection experiments, HD11 cells were infected with IBV at a multiplicity of infection (MOI) of 10 and harvested at the indicated time points for subsequent experiments.

### 2.2. Virus Infection and Ultraviolet Inactivation

HD11 cells were seeded into appropriate culture plates and infected with IBV at an MOI of 10 when cell confluence reached approximately 70–80%. After adsorption for 1 h at 37 °C, the virus inoculum was removed, and the cells were washed three times with phosphate-buffered saline (PBS). Fresh maintenance medium was then added, and cells were further cultured for the indicated times before sample collection. For ultraviolet (UV) inactivation, IBV-containing supernatants were collected and centrifuged at low speed to remove cell debris. The clarified supernatants were transferred to sterile culture dishes and exposed to UV irradiation in a biosafety cabinet for 4 h. Loss of viral infectivity after UV treatment was confirmed by TCID_50_ assay before subsequent experiments.

### 2.3. Cell Viability Assay

Cell viability was assessed using a Cell Counting Kit-8 (Beyotime, Shanghai, China, Catalogue No. C0038). HD11 cells were seeded into 96-well plates and subjected to the indicated treatments or IBV infection. At the indicated time points, 10 μL of CCK-8 reagent was added to each well and incubated at 37 °C for 1 h. Absorbance at 450 nm was measured using a microplate reader (Bio-Rad, Hercules, CA, USA). Cell viability was normalized to the mock group.

### 2.4. Cytotoxicity Assay

LDH release was detected using an LDH Cytotoxicity Assay Kit (Beyotime, Shanghai, China, Catalogue No. C0016). HD11 cells were seeded into 96-well plates and infected or treated as indicated. At the corresponding time points, cell culture supernatants were collected and mixed with LDH detection reagent in the dark at room temperature. Absorbance was measured at 490 nm using a microplate reader. LDH release was calculated as a percentage of total LDH release using the manufacturer’s recommended formula.

### 2.5. Measurement of Malondialdehyde Levels

Intracellular malondialdehyde (MDA) levels were determined using a Lipid Peroxidation MDA Assay Kit (Beyotime, Shanghai, China, Catalogue No. S0131S) according to the manufacturer’s instructions. HD11 cells were collected at the indicated time points after the corresponding treatments or IBV infection and lysed on ice. Cell lysates were centrifuged at 12,000× *g* for 10 min at 4 °C, and the supernatants were collected for analysis. The samples were mixed with the working reagent and heated in a boiling water bath for 15 min, followed by cooling to room temperature. Absorbance was measured at 532 nm using a microplate reader. MDA concentrations were calculated from a standard curve, normalized to the total protein concentration determined by the BCA assay, and expressed as μmol/mg protein.

### 2.6. Determination of Intracellular Glutathione Levels

The intracellular glutathione redox status was evaluated using a GSH and GSSG Assay Kit (Beyotime, Shanghai, China, Catalogue No. S0053) according to the manufacturer’s instructions. HD11 cells were collected after the indicated treatments or IBV infection and lysed on ice. Cell lysates were centrifuged at 10,000× *g* for 10 min at 4 °C, and the supernatants were collected for analysis. Total glutathione and oxidized glutathione (GSSG) were determined according to the manufacturer’s instructions. Absorbance was measured at 412 nm using a microplate reader. The intracellular reduced glutathione (GSH) level was calculated by subtracting twice the GSSG concentration from the total glutathione concentration, and the GSH/GSSG ratio was subsequently calculated according to the manufacturer’s instructions.

### 2.7. Detection of Intracellular Fe^2+^

Intracellular Fe^2+^ levels were measured using a Cellular Ferrous Iron Colorimetric Assay Kit (Wuhan Elabscience Biotechnology Co., Ltd., Wuhan, China, Catalogue No. E-BC-K881-M). HD11 cells were collected after the indicated treatments or IBV infection and lysed in lysis buffer at a ratio of 0.2 mL buffer per 1 × 10^6^ cells. After incubation on ice for 10 min, the lysates were centrifuged at 15,000× *g* for 10 min at 4 °C, and the supernatants were collected for subsequent analysis. Samples and standards were added to a 96-well plate, followed by addition of the chromogenic reagent according to the manufacturer’s protocol. After incubation at 37 °C for 10 min, absorbance at 593 nm was measured using a microplate reader. Intracellular Fe^2+^ levels were calculated according to the manufacturer’s instructions.

### 2.8. Transmission Electron Microscopy

HD11 cells were cultured to approximately 80–90% confluence and infected with IBV at an MOI of 10. At 36 h post-infection, cells were harvested by trypsinization and centrifuged at 1000 rpm for 5 min. The cell pellets were fixed with 2.5% glutaraldehyde prepared in 0.01 M phosphate-buffered saline (PBS) and kept at 4 °C for 5 min, followed by centrifugation at 12,000 rpm for 10 min. After removal of the supernatant, fresh fixative was carefully added along the tube wall, and the samples were stored at 4 °C before being submitted to Chengdu Lilai Biomedical Experimental Center (Chengdu, China) for subsequent TEM processing and imaging. TEM was used for qualitative ultrastructural assessment. Cells and mitochondria were examined across multiple microscopic fields, and representative images with clearly identifiable cellular and mitochondrial ultrastructure were selected for presentation. No predefined number of cells or mitochondria was used for quantitative analysis, and image evaluation was not performed in a blinded manner.

### 2.9. Quantitative Real-Time PCR and Viral RNA Copy Number Analysis

Total RNA was extracted from HD11 cells using TRIzol reagent (Invitrogen, Carlsbad, CA, USA). cDNA was synthesized using a reverse transcription kit (Takara, Dalian, China). Quantitative real-time PCR (qRT-PCR) was performed using gene-specific primers listed in Table 1. These primers were adopted from previously published studies [19,26]. Relative mRNA expression levels of ferroptosis-related genes, including *ACSL4*, *GPX4*, *SLC7A11* and *IREB2*, were normalized to *β-actin* and calculated using the 2^−ΔΔCT^ method. *β-actin* was selected as the reference gene based on its previous use in IBV-infected HD11 cells [19].

For viral RNA copy number analysis, infected cell cultures were subjected to freeze–thaw treatment and centrifuged, and viral RNA was extracted from the clarified supernatants using TRIzol reagent. Absolute quantitative qRT-PCR was performed using previously reported primers targeting the conserved 5′ untranslated region (5′UTR) of IBV (Table 1) and the previously described amplification conditions [27]. A recombinant plasmid containing the target fragment was used to generate the standard curve. Ten-fold serial dilutions of the standard plasmid ranging from 10^1^ to 10^8^ copies/μL were prepared, with each dilution tested in triplicate. Melting-curve analysis showed a single peak. The standard curve showed good linearity (R^2^ = 0.998), with a slope of −3.236 and an amplification efficiency of 103.7%. Viral RNA copy numbers were calculated according to the standard curve and expressed on the log10 scale. All qRT-PCR reactions were performed using SYBR qPCR Master Mix (Takara, Dalian, China) on a Bio-Rad real-time PCR system (Hercules, CA, USA).

### 2.10. Plasmid Construction and Transfection

The recombinant plasmid pcDNA3.1-Flag-N expressing the IBV nucleocapsid (N) protein was constructed and preserved in our laboratory, and successful expression of the N protein in HD11 cells was confirmed in our previous study [21,22]. HD11 cells were seeded into 6-well plates and transfected when cell confluence reached approximately 70–80%. Plasmid transfection was performed using Lipo8000 Transfection Reagent (Beyotime, Shanghai, China) according to the manufacturer’s instructions. Briefly, the plasmid and transfection reagent were separately diluted in serum-free DMEM and incubated for 5 min at room temperature before mixing. After incubation for an additional 15 min, the transfection mixture was added to the cells. Cells transfected with the empty pcDNA3.1 vector were used as the negative control. Samples were collected at the indicated time points for subsequent analyses.

### 2.11. Indirect Immunofluorescence Assay (IFA)

HD11 cells were fixed with 4% paraformaldehyde for 10 min after the indicated treatment. Cells were incubated with a mouse monoclonal antibody against IBV N protein (Novus Biologicals, Centennial, CO, USA; 1:200) for 1 h at 37 °C, followed by incubation with an FITC-conjugated secondary antibody (Sanying Biotech, Wuhan, China; 1:500) in the dark. Nuclei were stained with DAPI for 10 min, and fluorescence images were acquired using an Olympus IX71 fluorescence microscope (Olympus, Tokyo, Japan).

### 2.12. Pharmacological Treatments

Ferrostatin-1 (Fer-1) was purchased from MedChemExpress (Monmouth Junction, NJ, USA), RSL3 was obtained from AbMole (Houston, TX, USA), and epigallocatechin gallate (EGCG) was purchased from Macklin (Shanghai, China). HD11 cells were pretreated with the indicated compounds for 1 h before IBV infection. The concentrations of Fer-1, RSL3, and EGCG were selected with reference to concentrations used in previous studies [28,29,30] and were further verified in preliminary experiments under the present experimental conditions. Fer-1, EGCG, and RSL3 were used at final concentrations of 10 μM, 12.5 μM, and 2.5 μM, respectively. The final DMSO concentration was maintained at 0.02% in all corresponding treatment groups.

### 2.13. Statistical Analysis

Statistical analyses were performed using GraphPad Prism 8.0 software (GraphPad Software, San Diego, CA, USA). Data are presented as the mean ± SD of three independent biological experiments. Normality and homogeneity of variance were evaluated before parametric statistical analyses. Comparisons between two predefined groups were performed using Student’s *t*-test. For multiple-group comparisons, one-way ANOVA followed by Dunnett’s or Tukey’s multiple-comparisons test was used, as appropriate. For experiments involving both time and treatment as factors, two-way ANOVA followed by Šídák’s multiple-comparisons test was used. Viral titers and viral RNA copy numbers were analyzed on the log10 scale. A value of *p <* 0.05 was considered statistically significant. Statistical significance is indicated as follows: *p <* 0.05 (*) and *p <* 0.01 (**). The specific statistical tests used for individual experiments are indicated in the corresponding figure legends.

## 3. Results

### 3.1. IBV Induces Ferroptosis-Associated Changes in HD11 Cells

**Figure 1:**
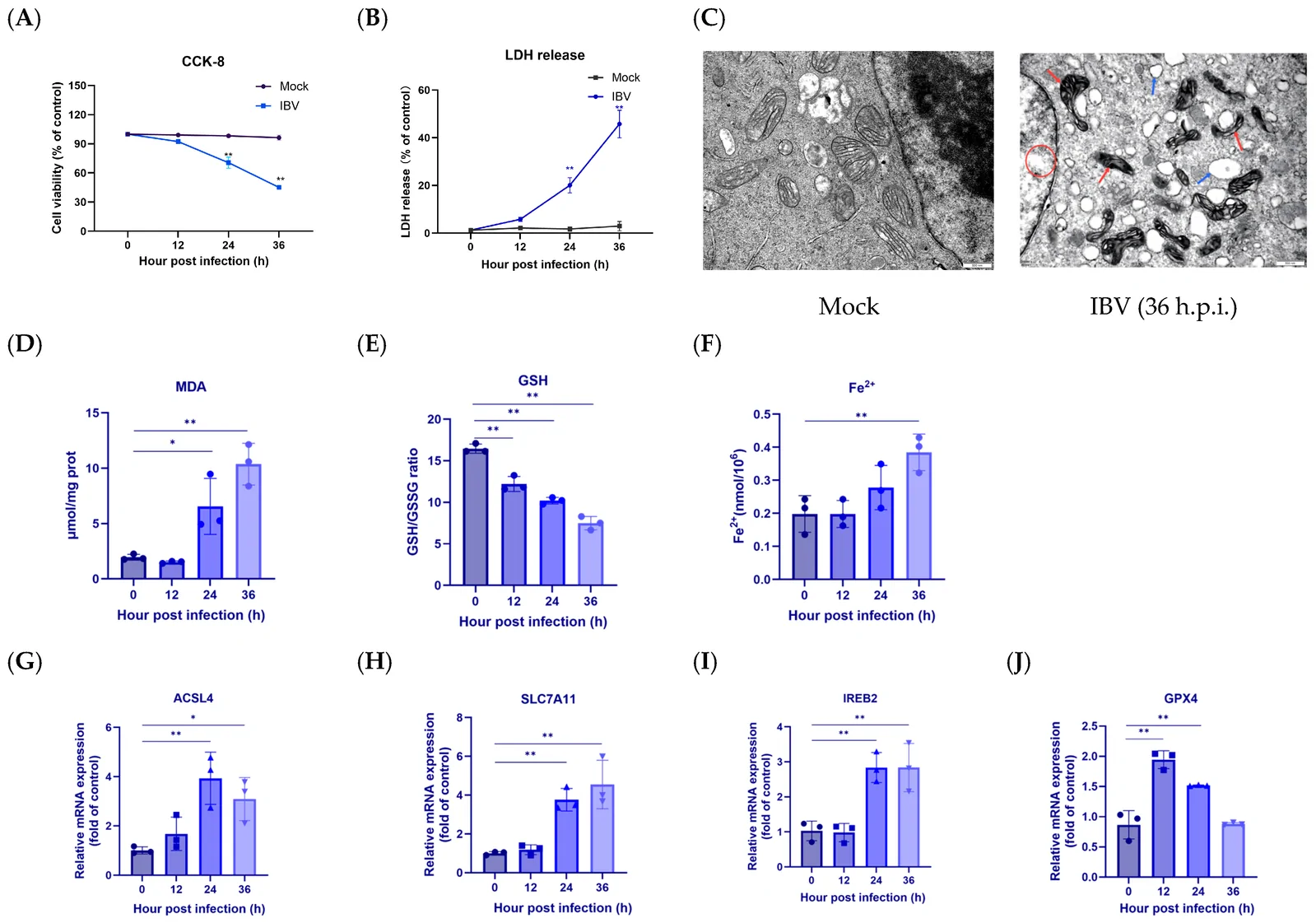
IBV infection induces ferroptosis-associated changes in HD11 cells. (**A**,**B**) Cell viability and LDH release in HD11 cells at the indicated time points after IBV infection. (**C**) Representative transmission electron microscopy images of HD11 cells. Red circles indicate altered nuclear morphology; red arrows indicate marked mitochondrial shrinkage; blue arrows indicate dilation of the rough endoplasmic reticulum. The TEM images were used for qualitative morphological assessment. Scale bars = 500 nm; original magnification, 25,000×. (**D**–**F**) Intracellular MDA levels, GSH/GSSG ratio, and Fe^2+^ levels in HD11 cells following IBV infection. (**G**–**J**) qRT-PCR analysis of *ACSL4*, *SLC7A11*, *IREB2*, and *GPX4* mRNA expression. Gene expression was normalized to *β-actin* relative to the control group. Each value is presented as the mean ± SD of three independent biological experiments. Panels (**A**,**B**) were analyzed by two-way ANOVA followed by Šídák’s multiple-comparisons test, whereas panels (**D**–**J**) were analyzed by one-way ANOVA followed by Dunnett’s multiple-comparisons test. * *p* < 0.05, ** *p* < 0.01.

IBV infection caused progressive injury in HD11 cells. Cell viability gradually decreased over time, whereas LDH release increased, indicating increased cell damage and membrane disruption (Figure 1A,B). TEM examination revealed ultrastructural alterations in IBV-infected HD11 cells, including mitochondrial shrinkage, altered nuclear morphology, and dilation of the rough endoplasmic reticulum (Figure 1C). Notably, mitochondrial shrinkage is consistent with the characteristic ultrastructural changes associated with ferroptosis. Consistent with these ultrastructural changes, IBV infection significantly increased MDA levels and progressively decreased the intracellular GSH/GSSG ratio, suggesting enhanced lipid peroxidation and redox imbalance (Figure 1D,E). Meanwhile, intracellular Fe^2+^ levels gradually increased following IBV infection (Figure 1F). qRT-PCR analysis showed that the ferroptosis-related genes *ACSL4*, *SLC7A11*, and *IREB2* were significantly upregulated after IBV infection. Their expression reached the highest levels at 24 h post-infection and remained elevated at 36 h (Figure 1G–I). In contrast, *GPX4* expression increased at 12 h post-infection and then gradually declined at the later time points (Figure 1J). These results indicate that IBV infection induces ferroptosis-associated metabolic, molecular, and ultrastructural changes in HD11 cells.

### 3.2. Fer-1 Attenuates IBV-Induced Ferroptosis-Associated Changes and Cellular Injury

**Figure 2:**
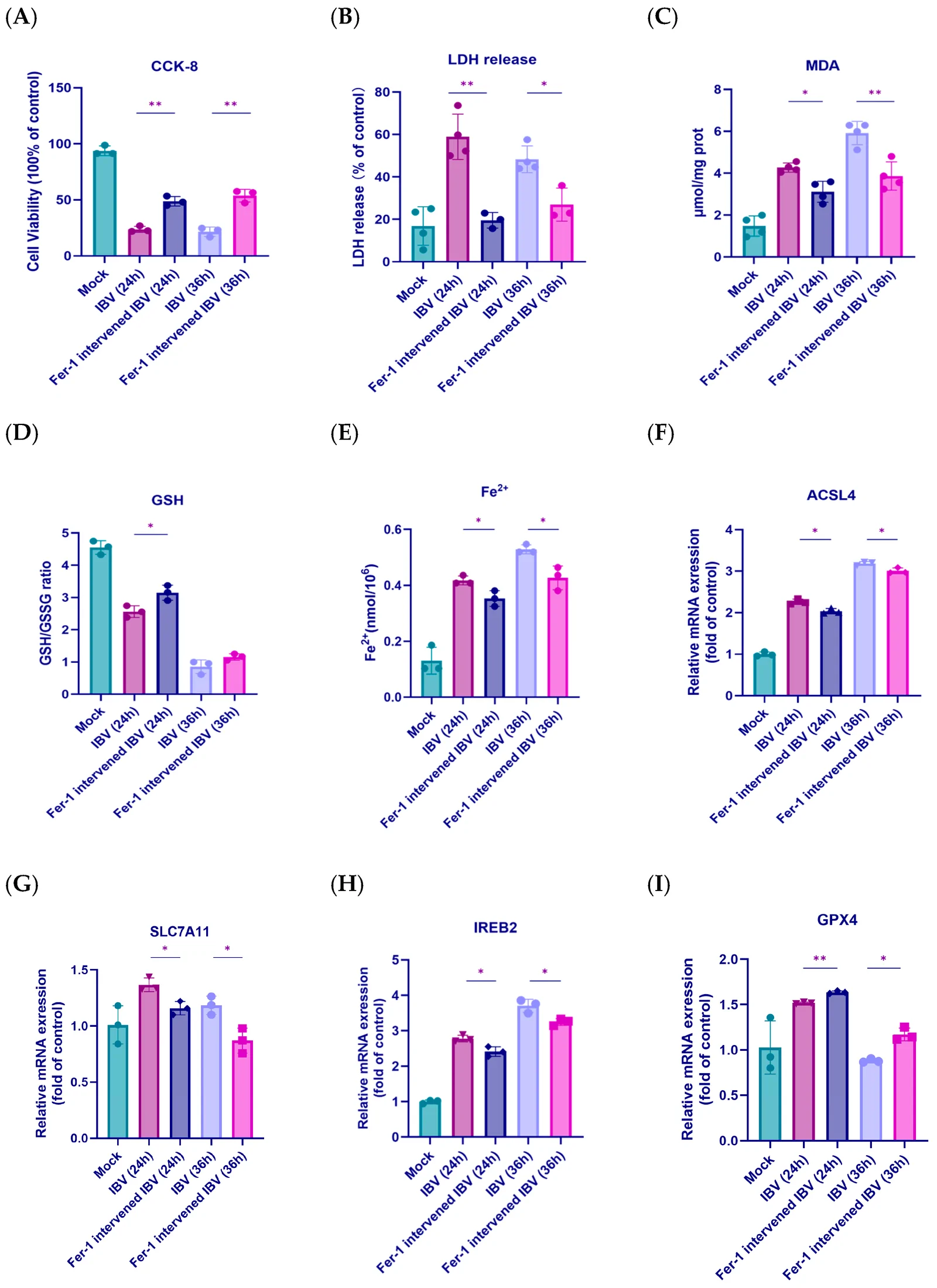
Fer-1 attenuates IBV-induced ferroptosis-associated cellular injury in HD11 cells. (**A**,**B**) Cell viability and LDH release in HD11 cells following IBV infection with or without Fer-1 treatment. (**C**–**E**) Intracellular MDA levels, GSH/GSSG ratio, and Fe^2+^ levels after the indicated treatments. (**F**–**I**) qRT-PCR analysis of *ACSL4*, *SLC7A11*, *IREB2*, and *GPX4* mRNA expression. HD11 cells were pretreated with Fer-1 (10 μM) for 1 h before IBV infection. Gene expression was normalized to *β-actin* relative to the control group. Each value is presented as the mean ± SD of three independent biological experiments. Statistical comparisons between the indicated groups were performed using Student’s *t*-test. * *p* < 0.05, ** *p* < 0.01.

To investigate the role of ferroptosis in IBV-induced cellular injury, HD11 cells were treated with the ferroptosis inhibitor ferrostatin-1 (Fer-1). Fer-1 showed no obvious cytotoxicity within the tested concentration range, and 10 μM was selected for subsequent experiments. IBV infection significantly reduced cell viability and increased LDH release, whereas Fer-1 pretreatment partially restored cell viability and decreased LDH leakage (Figure 2A,B). In addition, IBV infection markedly increased MDA levels and reduced the GSH/GSSG ratio, while these alterations were significantly reversed by Fer-1 treatment (Figure 2C,D). Intracellular Fe^2+^ accumulation induced by IBV infection was also markedly attenuated following Fer-1 treatment (Figure 2E). qRT-PCR analysis showed that IBV infection upregulated the expression of *ACSL4*, *SLC7A11*, and *IREB2*, whereas *GPX4* expression showed a time-dependent pattern, with an increase at 24 h and a slight decrease at 36 h post-infection, with these changes being partially reversed by Fer-1 treatment (Figure 2F–I). Together, these findings indicate that inhibition of ferroptosis alleviates IBV-induced cellular injury and ferroptosis-associated alterations in HD11 cells.

### 3.3. Replication-Competent IBV Is Associated with Ferroptosis-Associated Changes in HD11 Cells

**Figure 3:**
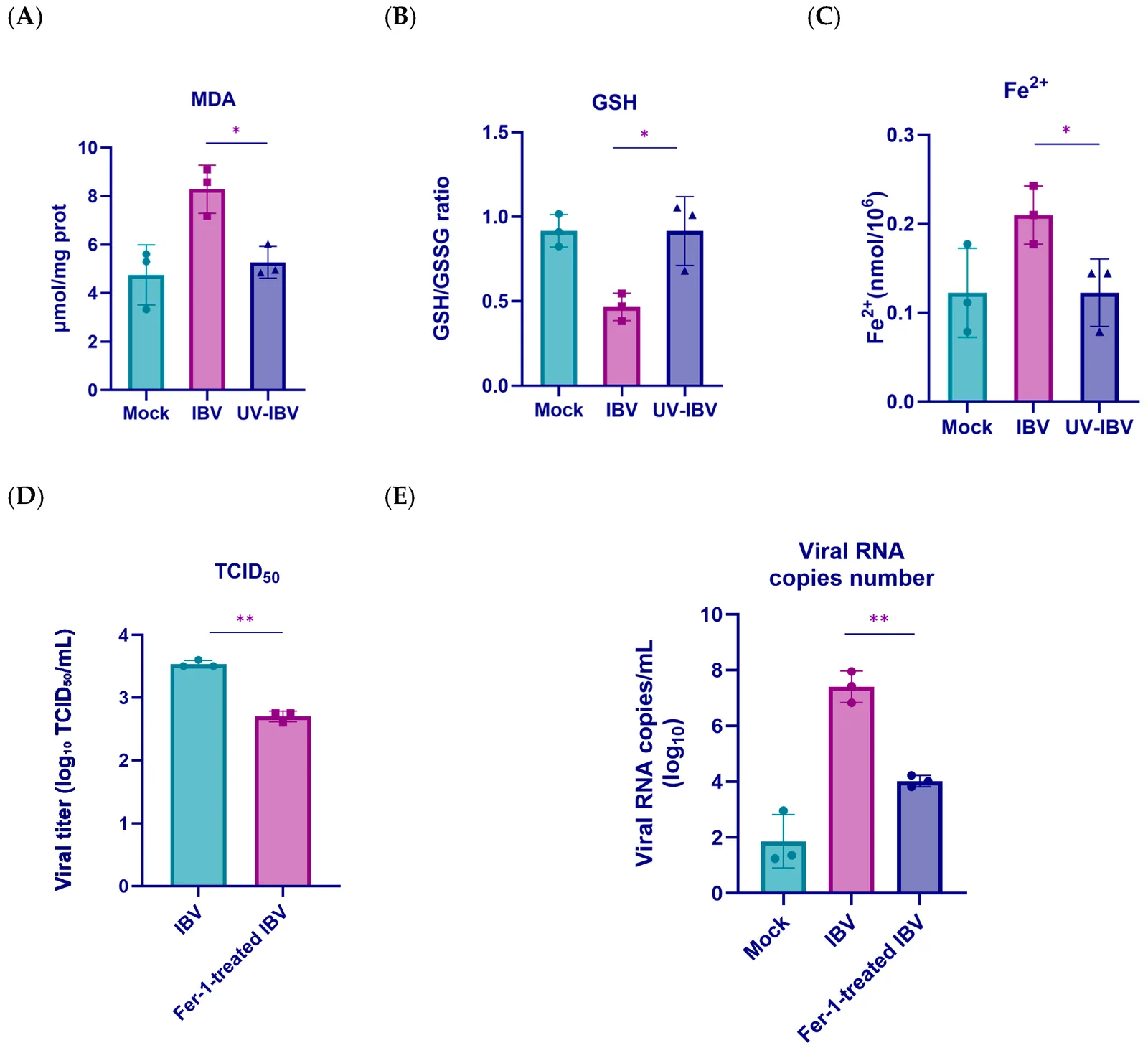
Replication-competent IBV, but not UV-inactivated IBV, was associated with ferroptosis-associated changes in HD11 cells under the conditions tested. (**A**–**C**) Intracellular MDA levels, GSH/GSSG ratio, and Fe^2+^ levels in HD11 cells infected with live IBV or UV-inactivated IBV. (**D**,**E**) Viral TCID_50_ titers and viral RNA copy numbers in IBV-infected HD11 cells with or without Fer-1 treatment. Each value is presented as the mean ± SD of three independent biological experiments. Statistical comparisons between the indicated groups were performed using Student’s *t*-test. * *p* < 0.05, ** *p* < 0.01.

To determine whether IBV-induced ferroptosis depends on viral replication, IBV was inactivated by ultraviolet (UV) irradiation before infection. TCID_50_ analysis confirmed complete loss of infectivity in the UV-inactivated group, as no detectable viral titers were observed. Compared with the mock and UV-inactivated groups, infection with replication-competent IBV significantly increased MDA levels and reduced the intracellular GSH/GSSG ratio (Figure 3A,B). Intracellular Fe^2+^ accumulation was also markedly elevated only in cells infected with live IBV but not in those treated with UV-inactivated virus (Figure 3C), indicating that ferroptosis-associated metabolic alterations depend on active viral replication. Fer-1 treatment significantly reduced infectious viral titers measured by TCID_50_ (Figure 3D) and viral RNA copy numbers measured by absolute qRT-PCR (Figure 3E). These results indicate that ferroptosis inhibition is associated with reduced viral output in IBV-infected HD11 cells. Together, these findings indicate that replication-competent IBV is associated with ferroptosis-associated changes and that inhibition of these changes by Fer-1 is accompanied by reduced viral replication in HD11 cells.

### 3.4. IBV N Protein Induces Ferroptosis-Associated Changes in HD11 Cells

**Figure 4:**
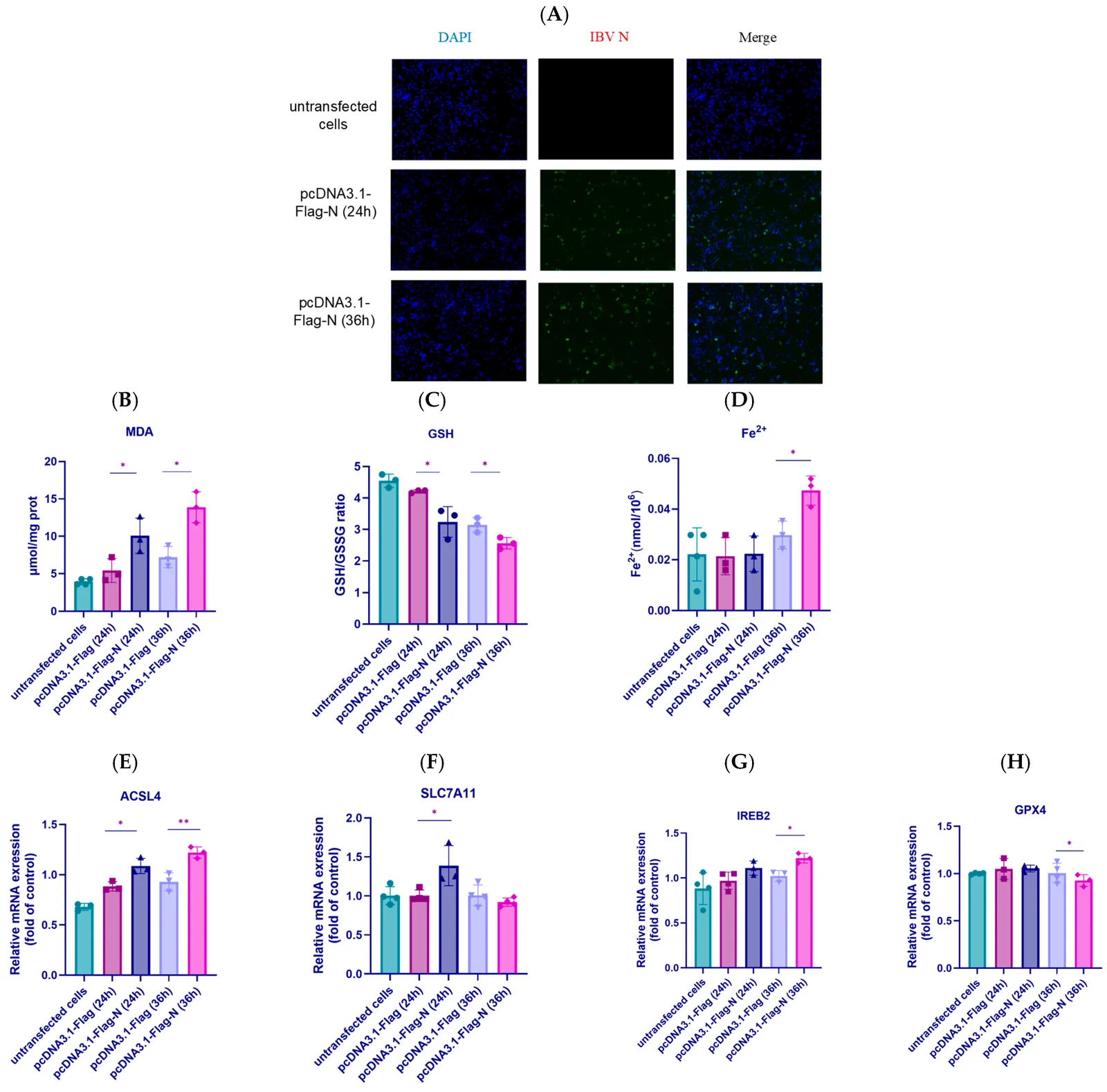
IBV nucleocapsid (N) protein induces ferroptosis-associated changes in HD11 cells. (**A**) Immunofluorescence confirmation of N protein expression in HD11 cells at 24 and 36 h after transfection with the pcDNA3.1-Flag-N plasmid; untransfected cells were included as the negative control for N-protein staining. (**B**–**D**) Intracellular MDA levels, GSH/GSSG ratio, and Fe^2+^ levels following N protein expression. (**E**–**H**) qRT-PCR analysis of *ACSL4*, *SLC7A11*, *IREB2*, and *GPX4* mRNA expression. For panels (**B**–**H**), cells transfected with the empty vector were used as the corresponding controls. Gene expression was normalized to *β-actin* and expressed relative to the empty vector control group. Data are presented as the mean ± SD of three independent biological experiments. For panels (**B**–**H**), N-transfected cells were compared with the corresponding empty-vector controls at each time point using Student’s *t*-test. * *p* < 0.05, ** *p* < 0.01.

To determine whether the IBV nucleocapsid (N) protein contributes to ferroptosis induction, HD11 cells were transfected with the pcDNA3.1-Flag-N plasmid. Immunofluorescence analysis confirmed N protein expression in HD11 cells at both 24 and 36 h after transfection, whereas no specific fluorescence signal was observed in untransfected cells (Figure 4A). N protein expression significantly increased MDA levels in a time-dependent manner (Figure 4B). Meanwhile, the intracellular GSH/GSSG ratio progressively decreased following N protein expression (Figure 4C). Intracellular Fe^2+^ levels were also significantly increased in N protein-expressing cells compared with the empty vector control (Figure 4D). qRT-PCR analysis showed that *ACSL4* expression was significantly increased at both 24 and 36 h after transfection. *SLC7A11* expression was transiently upregulated at 24 h but returned to the control level at 36 h. At 36 h after transfection, *IREB2* expression was significantly increased, whereas *GPX4* expression was significantly decreased (Figure 4E–H). These findings indicate that IBV N-protein expression can reproduce several ferroptosis-associated changes in HD11 cells.

### 3.5. EGCG Attenuates Ferroptosis-Associated Injury in IBV-Infected HD11 Cells

**Figure 5:**
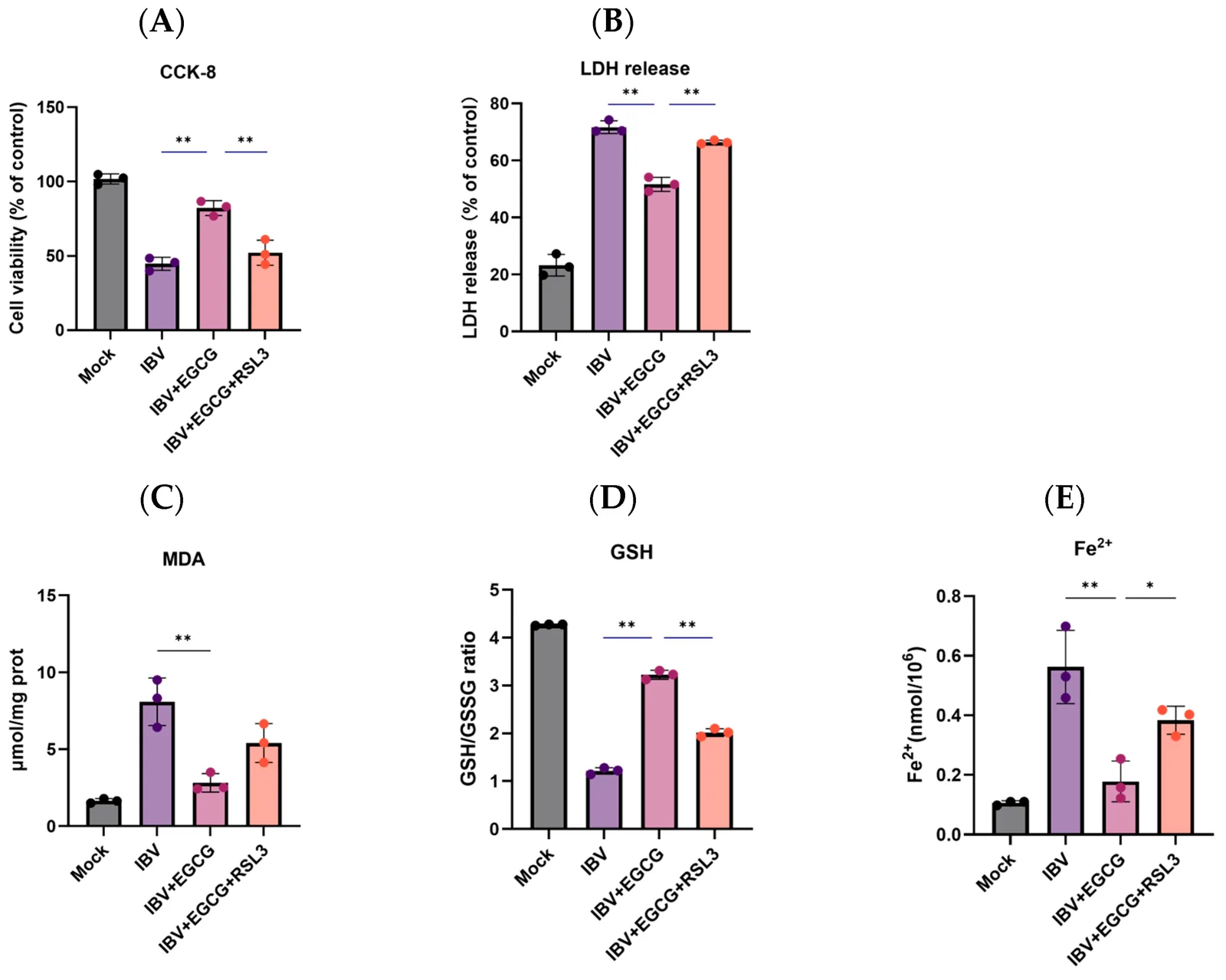
EGCG attenuates IBV-induced ferroptosis-associated cellular injury in HD11 cells. (**A**,**B**) Cell viability and LDH release in HD11 cells following IBV infection with EGCG treatment in the presence or absence of RSL3. (**C**–**E**) Intracellular MDA levels, GSH/GSSG ratio, and Fe^2+^ levels after the indicated treatments. HD11 cells were pretreated with EGCG (12.5 μM) or RSL3 (2.5 μM) for 1 h before IBV infection. Each value is presented as the mean ± SD of three independent biological experiments. Statistical analysis was performed using one-way ANOVA followed by Tukey’s multiple-comparisons test. * *p* < 0.05, ** *p* < 0.01.

To further investigate the protective effect of epigallocatechin gallate (EGCG) against IBV-induced ferroptosis-associated cellular injury, HD11 cells were treated with EGCG (12.5 μM) in the presence or absence of the ferroptosis inducer RSL3 (2.5 μM). IBV infection significantly reduced cell viability and increased LDH release. EGCG treatment partially restored cell viability and decreased LDH release. These effects were partially reversed by RSL3 co-treatment (Figure 5A,B). EGCG also significantly reduced MDA levels and restored the intracellular GSH/GSSG ratio in IBV-infected cells (Figure 5C,D). Furthermore, EGCG markedly decreased IBV-induced intracellular Fe^2+^ accumulation, whereas RSL3 partially reversed this effect (Figure 5E). These results indicate that EGCG protects against IBV-induced ferroptosis-associated cellular injury in HD11 cells, whereas RSL3 treatment partially reverses this protective effect.

## 4. Discussion

Ferroptosis is an important pathological mechanism involved in virus-induced cellular injury and has been increasingly recognized as a critical factor affecting virus–host interactions [9]. Previous studies have shown that several viruses can induce ferroptosis-associated metabolic disorders, including lipid peroxidation, glutathione depletion, and iron dysregulation [31,32]. Hepatitis C virus (HCV) infection promotes iron accumulation and oxidative damage in hepatocytes, contributing to liver injury [33]. Influenza A virus (IAV) infection induces ferroptosis-associated lung injury through excessive oxidative stress and abnormal iron metabolism [34]. In addition, SARS-CoV-2 infection has been reported to induce ferroptosis-like changes characterized by reduced antioxidant capacity, increased lipid peroxidation, and disturbed iron homeostasis [35]. Previous studies have shown that IBV infection can activate endoplasmic reticulum stress and apoptosis, highlighting the involvement of cellular stress responses in IBV-induced cell injury [36]. However, whether ferroptosis is involved in IBV infection remains unclear. In the present study, we demonstrated that IBV infection induced typical ferroptosis-associated alterations in HD11 chicken macrophages, including increased MDA levels, decreased GSH/GSSG ratio, intracellular Fe^2+^ accumulation, and mitochondrial damage. The TEM observations in the present study were qualitative and were not subjected to quantitative morphometric analysis. Furthermore, IBV infection altered the expression of ferroptosis-related genes. ACSL4 promotes the incorporation of polyunsaturated fatty acids into membrane phospholipids and thereby increases cellular susceptibility to lipid peroxidation, whereas GPX4 is an important antioxidant enzyme that limits phospholipid peroxidation [37,38]. IREB2 is involved in the regulation of intracellular iron homeostasis and has also been associated with ferroptosis sensitivity [10]. In contrast to the canonical ferroptosis pattern, SLC7A11 expression was increased after IBV infection. Since SLC7A11 supports cystine uptake and GSH synthesis, this upregulation may represent a compensatory response to infection-induced oxidative stress [10]. However, this response appeared insufficient to prevent the decrease in the GSH/GSSG ratio and GPX4 expression at the late stage of infection. Taken together, these results suggest that IBV infection disturbs redox and iron homeostasis and involves ferroptosis-associated processes in chicken macrophages, although the underlying regulatory events remain to be further characterized.

Ferroptosis inhibition has been shown to protect cells from virus-induced oxidative damage and cellular injury [39,40]. Ferrostatin-1 (Fer-1) is a radical-trapping antioxidant widely used to inhibit ferroptosis by suppressing lipid peroxidation [41,42]. Previous studies have demonstrated that pharmacological inhibition of ferroptosis can reduce lipid peroxidation, restore redox balance, and improve cell survival in several viral infection models. For example, ferrostatin-1 treatment reduces ferroptosis-associated neuronal injury and improves cell survival during enterovirus A71 infection [39]. Similar findings have also been reported in severe fever with thrombocytopenia syndrome virus (SFTSV) infection, in which virus-induced ferroptosis contributes to tissue injury, suggesting that targeting ferroptosis may reduce virus-induced cellular damage [40]. In the present study, Fer-1 treatment reduced IBV-induced cellular injury. The increased cell viability and decreased LDH release following Fer-1 treatment reflected reduced cellular injury, while the reductions in MDA and Fe^2+^ levels and restoration of the GSH/GSSG ratio were consistent with attenuation of ferroptosis-associated changes. These findings suggest that ferroptosis-associated processes are involved in IBV-induced cellular damage. In addition, Fer-1 partially reversed the IBV-induced dysregulation of ferroptosis-related genes, including *ACSL4*, *SLC7A11*, *IREB2*, and *GPX4*, further supporting that ferroptosis is actively involved in the cellular response to IBV infection. These findings support ferroptosis as one component of IBV-induced cellular injury. Previous studies have shown that IBV infection can also induce apoptosis and pyroptosis in HD11 cells [21,22], indicating that multiple regulated cell-death pathways may coexist during IBV infection. Therefore, the ferroptosis-associated changes observed in the present study do not exclude the involvement of these previously identified cell-death pathways.

Our results further suggest a close relationship between active IBV infection, ferroptosis, and viral replication. In contrast to replication-competent IBV, UV-inactivated IBV did not induce comparable changes in MDA, GSH/GSSG, or intracellular Fe^2+^ levels, supporting an association between active IBV infection and these ferroptosis-associated responses under the conditions tested. Moreover, Fer-1 treatment reduced both IBV RNA copy numbers and infectious viral titers. Thus, inhibition of ferroptosis-associated changes by Fer-1 was accompanied by reduced IBV replication. However, Fer-1 is a radical-trapping antioxidant that suppresses lipid peroxidation, and its effects on cellular lipid oxidation may also influence membrane-dependent processes involved in the viral life cycle [41,42]. Therefore, the present findings do not establish that ferroptosis directly promotes IBV replication. Other host cellular processes have also been linked to IBV replication. For example, IBV-induced autophagy has been reported to facilitate viral replication through activation of the VPS34 complex [43]. Similar relationships between ferroptosis and viral replication have been reported for other viruses [44,45]. For example, influenza A virus (IAV) has been shown to exploit ferroptotic responses induced by its hemagglutinin protein, thereby creating a cellular environment favorable for viral replication. Similarly, severe fever with thrombocytopenia syndrome virus (SFTSV) promotes ferroptosis through m6A-related ferritinophagy, and pharmacological inhibition of ferroptosis effectively suppresses viral replication and tissue injury [40,46]. These observations suggest a close relationship between ferroptosis-associated changes and IBV replication, although how these changes influence the viral replication process remains to be further elucidated.

Coronavirus nucleocapsid (N) proteins have been increasingly recognized as multifunctional proteins that regulate host cellular responses in addition to their essential roles in viral replication and genome packaging [47,48]. For example, the SARS-CoV-2 N protein has been reported to interfere with RIG-I/MDA5-dependent interferon signaling and reduce antiviral responses [49]. Coronavirus N proteins have also been reported to regulate inflammatory signaling and programmed cell death. The SARS-CoV N protein induces apoptosis and activates inflammatory responses, further supporting the multifunctional roles of coronavirus N proteins in regulating host cell fate [50]. For IBV, the N protein has been shown to interact with multiple host cellular proteins involved in translation, RNA processing, and cellular stress responses, supporting its broader role in virus–host interactions [51]. In our previous study, we demonstrated that expression of the IBV N protein alone was sufficient to activate the NLRP3 inflammasome and induce pyroptosis in HD11 cells [21]. Consistent with our previous findings, we further demonstrated that expression of the IBV N protein induced typical ferroptosis-associated alterations, including increased MDA levels, a reduced GSH/GSSG ratio, intracellular Fe^2+^ accumulation, and dysregulated expression of ferroptosis-related genes. These findings indicate that the IBV N protein alone is sufficient to trigger ferroptosis-associated responses in the absence of complete viral infection. Together with our previous findings, the present results suggest that the IBV N protein may function as an important regulator of multiple programmed cell death pathways during IBV infection. Future studies are needed to determine whether the IBV N protein directly regulates iron metabolism, lipid metabolism, or redox homeostasis to initiate ferroptosis. Although IBV N protein expression induced ferroptosis-associated changes in HD11 cells, the underlying mechanism remains unclear. Previous studies have shown that the IBV N protein interacts with multiple host proteins and modulates host cellular processes [51,52]. In addition, a Fer-1 rescue experiment was not performed in the N-protein expression model; therefore, the observed changes should be interpreted as ferroptosis-associated responses rather than direct evidence that the N protein specifically induces ferroptosis. Whether the observed changes result from direct regulation of ferroptosis-related pathways or indirectly from cellular stress associated with N protein expression requires further investigation.

**Figure 6:**
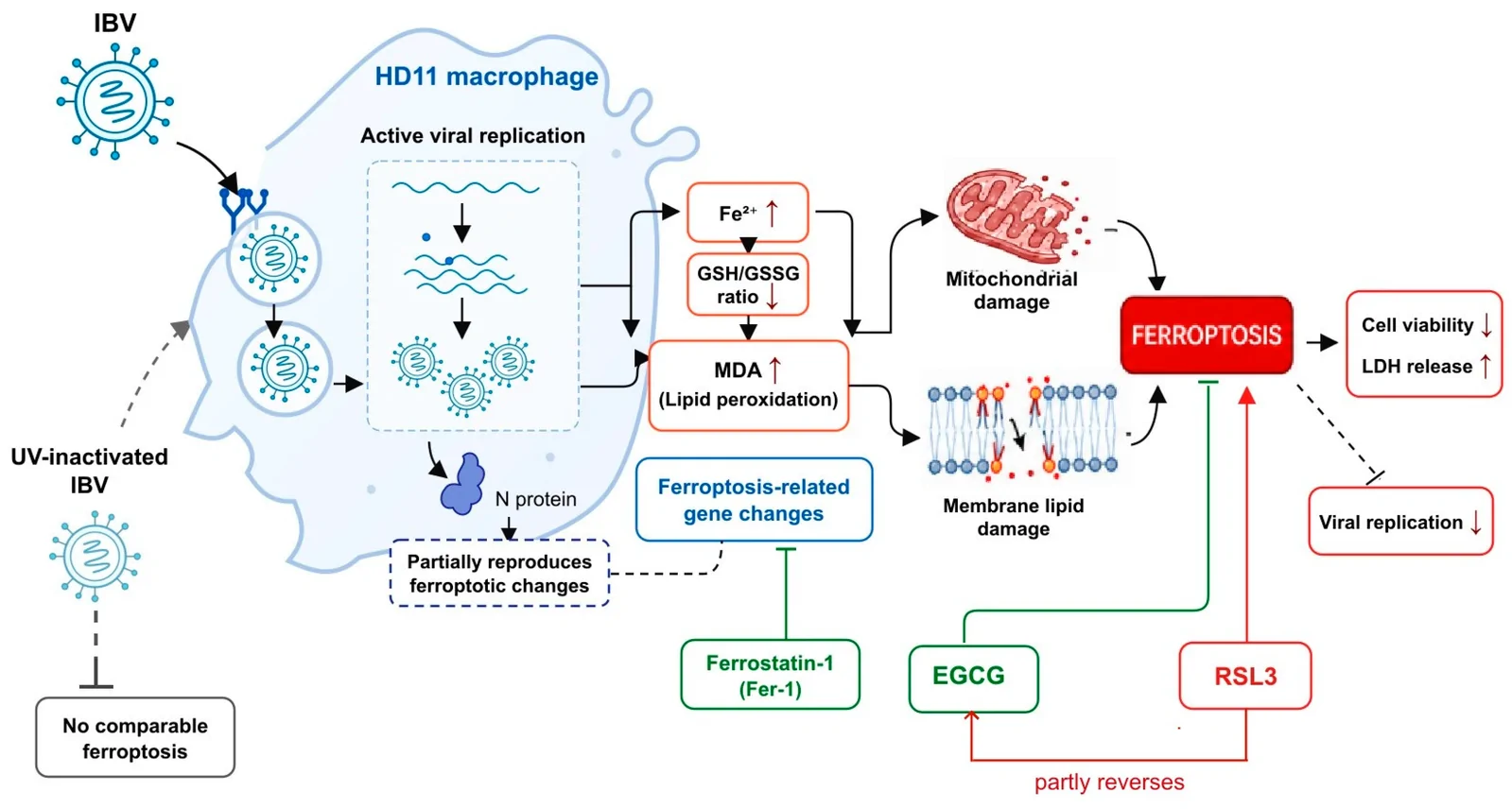
IBV-induced ferroptosis and viral replication in HD11 chicken macrophages. Active IBV replication and the N protein induce ferroptosis-associated changes, while ferroptosis is associated with cellular injury and viral replication. Fer-1 and EGCG inhibit ferroptosis, whereas RSL3 partially reverses the protective effect of EGCG. Upward and downward arrows indicate increased and decreased levels, respectively.

Because ferroptosis was associated with IBV-induced cellular injury, we further examined whether modulation of this process could protect infected cells. Epigallocatechin gallate (EGCG), a natural polyphenolic compound derived from green tea, has been reported to exhibit antioxidant and antiviral activities in several viral infection models [53,54]. For example, EGCG inhibits influenza A virus (IAV) infection by reducing viral entry and suppressing oxidative stress [55]. In Japanese encephalitis virus (JEV) infection, EGCG attenuates oxidative damage and inflammatory responses by restoring intracellular redox homeostasis [56]. In addition, EGCG has recently been shown to decrease ferroptosis-associated injury by reducing lipid peroxidation and improving antioxidant capacity [57]. In the present study, EGCG treatment significantly reduced IBV-induced ferroptotic responses, as indicated by decreased MDA and Fe^2+^ accumulation, restoration of the intracellular GSH/GSSG ratio, and improved cell viability. Moreover, the protective effects of EGCG were partially reversed by RSL3 treatment, suggesting that modulation of ferroptosis contributes, at least in part, to EGCG-mediated protection. However, given the broad antioxidant properties of EGCG, other mechanisms may also contribute to its protective effects against IBV-induced cellular injury. Taken together, the proposed relationships among IBV infection, ferroptosis-associated cellular injury, viral replication, and the effects of Fer-1 and EGCG are summarized in Figure 6.

The present findings should be interpreted in the context of several methodological considerations. Lipid peroxidation was evaluated primarily by MDA, and the expression of *GPX4*, *ACSL4*, and *SLC7A11* was assessed at the transcriptional level; complementary live-cell lipid ROS measurements and protein-level analyses were beyond the scope of the present study. In addition, because Fer-1 acts as a radical-trapping antioxidant, its broader effects on cellular lipid oxidation and membrane-related processes should be considered when interpreting the association between Fer-1 treatment and reduced viral replication. Although HD11 cells provide a useful model for investigating IBV-macrophage interactions, the use of a single transformed macrophage cell line represents a limitation of the present study. Ferroptosis-associated responses may differ among cell types, particularly in epithelial cells of the respiratory, renal, and reproductive systems that are major targets of IBV during natural infection [1,3]. In addition, the relatively high MOI of 10 used in this study may not fully reflect the viral exposure encountered during natural infection and should be considered when interpreting the biological relevance of the findings. Further studies using primary chicken macrophages, relevant epithelial cells, and in vivo models will be important to determine the broader relevance of ferroptosis to IBV pathogenesis.

## 5. Conclusions

In conclusion, this study identifies ferroptosis-associated changes as an important component of the cellular response to IBV infection in chicken macrophages, with these changes occurring in association with viral replication. The findings also suggest that the IBV N protein contributes to ferroptosis-associated cellular changes, providing further insight into the interaction between IBV and host cell death pathways. These findings provide further insight into the involvement of ferroptosis-associated processes in IBV-induced cellular injury and warrant further investigation.

## Figures and Tables

**Table 1:** qRT-PCR primer sequences.

Gene	Forward Primer (5′–3′)	Reverse Primer (5′–3′)
*ACSL4*	GCTAAGCCCATCTCGGACAAACC	CCAACGTGTCTGCTCCAGGAATG
*SLC7A11*	GCTGTCGTGACGGTGCCTAATG	CTCTTGTGGCTGCCTGCTGTC
*IREB2*	GTCTATCACCAGGCAGCGGAATG	ACCCATAACCAACAACCTCAAACCC
*GPX4*	GCTGTGGAAGTGGCTGAAGGAG	TTGGCCCTCACGGTTGATAAGAAAC
*β-actin*	CCAGCCATGTATGTAGCCATCCAG	GGTAACACCATCACCAGAGTCCATC
*IBV-5’UTR*	ACAGGTTCTGGTGGTGTTTAGTGA	AGTTGTTCGGGAATGTCTTTGG

## Data Availability

The original contributions presented in this study are included in the article. Further inquiries can be directed to the corresponding author.
